# Deep Learning–Based Image Analysis of Liver Steatosis in Mouse Models

**DOI:** 10.1016/j.ajpath.2023.04.014

**Published:** 2023-05-24

**Authors:** Laura Mairinoja, Hanna Heikelä, Sami Blom, Darshan Kumar, Anna Knuuttila, Sonja Boyd, Nelli Sjöblom, Eva-Maria Birkman, Petteri Rinne, Pekka Ruusuvuori, Leena Strauss, Matti Poutanen

**Affiliations:** ∗ Research Centre for Integrative Physiology and Pharmacology, Institute of Biomedicine and Turku Center for Disease Modeling, University of Turku, Turku, Finland; † Aiforia Technologies Oyj, Helsinki, Finland; ‡ Department of Pathology, University of Helsinki and Helsinki University Hospital, Helsinki, Finland; § Department of Pathology, Turku University Hospital and University of Turku, Turku, Finland; ¶ Cancer Research Unit, Institute of Biomedicine, University of Turku, Turku, Finland; ‖ Department of Internal Medicine and Clinical Nutrition, Centre for Bone and Arthritis Research, Institute of Medicine, The Sahlgrenska Academy, University of Gothenburg, Gothenburg, Sweden

## Abstract

The incidence of nonalcoholic fatty liver disease is a continuously growing health problem worldwide, along with obesity. Therefore, novel methods to both efficiently study the manifestation of nonalcoholic fatty liver disease and to analyze drug efficacy in preclinical models are needed. The present study developed a deep neural network–based model to quantify microvesicular and macrovesicular steatosis in the liver on hematoxylin-eosin–stained whole slide images, using the cloud-based platform, Aiforia Create. The training data included a total of 101 whole slide images from dietary interventions of wild-type mice and from two genetically modified mouse models with steatosis. The algorithm was trained for the following: to detect liver parenchyma, to exclude the blood vessels and any artefacts generated during tissue processing and image acquisition, to recognize and differentiate the areas of microvesicular and macrovesicular steatosis, and to quantify the recognized tissue area. The results of the image analysis replicated well the evaluation by expert pathologists and correlated well with the liver fat content measured by EchoMRI *ex vivo*, and the correlation with total liver triglycerides was notable. In conclusion, the developed deep learning–based model is a novel tool for studying liver steatosis in mouse models on paraffin sections and, thus, can facilitate reliable quantification of the amount of steatosis in large preclinical study cohorts.

## Graphical abstract

**Figure:**
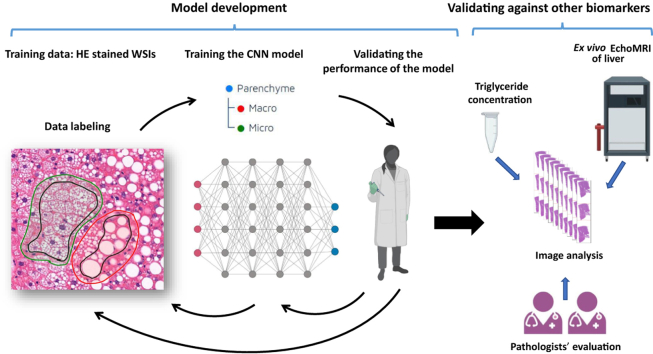

Nonalcoholic fatty liver disease (NAFLD) is the hepatic manifestation of metabolic syndrome.^1^ The prevalence of NAFLD (eg, nonalcoholic steatosis, steatohepatitis, and liver fibrosis) is increasing worldwide alongside lifestyle-related diseases, such as obesity and diabetes, leading to increased liver-related morbidity and mortality.^1^ The hallmarks of NAFLD are microvesicular and macrovesicular lipid droplets accumulating inside the hepatocytes.^2^ Microvesicular steatosis is characterized by the presence of small fat droplets with preserved cellular architecture, whereas in macrovesicular steatosis, large droplets that displace the nucleus are formed.^3^^,^^4^ Microvesicular steatosis is associated with higher grades of steatosis,^5^ and when present it may indicate increased disease severity or drug-induced hepatotoxity.^6^

The type and amount of liver steatosis is a central quantifiable phenotype in experimental mouse models of NAFLD, frequently utilized in studies aimed at understanding the pathogenesis of disease or exploring new therapeutic options. Traditionally, the analysis of histopathology, such as the evaluation of liver steatosis, has been based on pathologist's visual evaluation, as computational analysis of images has only fairly recently become an option in histopathology.^7^ During the past 5 years, deep learning (DL)–based methods have become popular in such image analysis, because of the development of graphics processing units and more powerful memory resources.^8^ Higher computer power is needed in fields such as histology, which has a high amount of digitized data.^9^ DL is a machine learning method that can computationally learn image features for digitized image analysis. DL is based on artificial neural networks that are composed of layers of nodes. In image analysis, convolutional neural networks are the most commonly used DL method. In convolutional neural networks, the convolution operation is used at least on one of the layers of the deep neural network.^10^

Histology is the reference standard for staging NAFLD severity.^11^ A widely used histologic scoring for liver steatosis is called the Kleiner score, where the histopathologic features of NAFLD, and its severe form, nonalcoholic steatohepatitis, are assessed.^12^ Many novel deep learning models are based on detecting and quantifying these histologic features, which have traditionally been evaluated by pathologists. One approach is to use hematoxylin-eosin (HE)–stained liver tissue sections for generating deep learning models to detect lipid droplets,^13^^,^^14^ inflammation, ballooning, and collagen fibers.^15^ Prior work in developing deep learning models to detect fat accumulation in the liver has concentrated on segmenting and quantifying macrovesicular lipid droplets.^13^^,^^14^ However, there has been less attention on developing methods to quantify microvesicular steatosis, although a few automatic methods have been published.^16^ The task of quantifying microvesicular steatosis poses a challenge for automated image analysis, because the size and shape of lipid droplets vary and the discrimination of microvesicular steatosis from general ballooning is challenging.^17^ However, quantifying both microvesicular and macrovesicular steatosis is essential to better define the stage of the steatosis in the experimental models.

This study developed a deep neural network–based model in Aiforia Create that recognizes and differentiates microvesicular and macrovesicular steatosis in the HE-stained whole slide images (WSIs) of mouse liver tissue sections. The DL model measured the tissue areas in the section covered by these features and provided a reliable tool to obtain a comprehensive analysis of the severity of liver steatosis in an experimental mouse model of NAFLD. The study validated the image analysis result with two experienced human pathologists (S.B. and N.S.), as well as two independent methods of lipid content measurement.

## Materials and Methods

### Mouse Models

All animal work was conducted at the Turku Center for Disease Modeling, University of Turku (Turku, Finland), under the animal license numbers 10605/04.10.07/2016 and 41729/2019, granted by the Animal Experiment Board in Finland. Animal handling was performed in accordance with the institutional animal care policies that fully meet the requirements as defined in the NIH guidelines on animal experimentation.^18^

Animals were housed in individually ventilated cages (Techniplast, Buguggiate, Italy) with approximately 70 air changes per hour. Constant temperature (21°C ± 3°C) and humidity (55% ± 15%) were maintained together with a consistent 12-hour light-dark cycle, with a light change at 7 am and 7 pm. Autoclaved aspen chips were used as bedding (Tapvei Ltd, Harjumaa, Estonia). The chow and water were available *ad libitum.* The genetically modified mice and their littermates were individually identified with earmarks and housed with littermates, one to six mice per cage. The genotyping protocols applied have been documented in previous publications.^19^^,^^20^

For training the DL model, the authors used mouse models, which had been previously characterized to present with liver steatosis. The training data included HE-stained WSIs of formalin-fixed, paraffin-embedded mouse liver samples from 9-month–old mice deficient in hydroxysteroid (17β) dehydrogenase 13^19^ or deficient in hydroxysteroid (17β) dehydrogenase 12,^20^ and their wild-type littermates in C57Bl6; 129S5 hybrid (50/50) and C57BL/6NCrl genetic background, respectively. In addition, the training data included HE-stained WSIs of mouse liver samples obtained from C57Bl wild-type mice kept on a Western diet (D12079B; Research Diets Inc., New Brunswick, NJ) for 14 weeks. Validation data included HE-stained WSIs of mouse liver samples from wild-type mice with C57Bl6; 129 hybrid genetic background and mice with same background kept in high-fat diet (HFD) conditions (D17010103; Research Diets Inc.)^21^ for 12 weeks.

### Liver Tissue Composition, Triglyceride Content, and Histologic Analysis

Mice were euthanized using CO_2_ asphyxiation followed by cervical dislocation. Liver tissue was dissected, and the amount of total fat, free water, and lean mass was measured from the whole liver with an EchoMRI-700 device (Echo Medical Systems, Houston, TX).

For the histology, the medial lobe of the liver was collected, and tissue samples were fixed in 10% formalin for 24 to 48 hours, dehydrated, and embedded in paraffin. Sections (4 μm thick) were cut and stained with HE following standard procedures.

Hepatic triglycerides were measured with a GPO-PAP kit (Mti Diagnostics, Idstein, Germany), following the manufacturer's instructions. Frozen liver samples of 100 mg were homogenized in 500 μL of 0.1% Triton X-100 in phosphate-buffered saline with Tissue Lyser (Qiagen, Hilden, Germany) for 1.5 minutes, followed by centrifugation to remove any insoluble material. Supernatants were collected and diluted 1:4 in 0.1% Triton X-100 in phosphate-buffered saline before measurement. The absorbance (540 nm) was measured with EnSight multimode plate reader (PerkinElmer, Waltham, MA).

### Imaging and Software

To generate the WSIs, the HE-stained microscope slides were scanned using a Pannoramic 250 and 1000 Flash digital slide scanners (3DHISTECH Ltd, Budapest, Hungary) with ×20 magnification with a resolution of 0.24 μm/pixel. The image quality was ensured by the automatic scanner initialization and visual inspection after scanning (ie, verifying that the images are in focus and no artefacts are present). The WSIs were converted from an MRXL format to an ECW format and uploaded into Aiforia Create, an image management and analysis cloud platform version 5.2 (Aiforia Technologies, Helsinki, Finland), which aids the development of a machine learning models with deep convolutional neural networks and supervised learning. After the conversion, image quality was verified to remain the same with visual verification.

### Generating a Model to Quantify Hepatic Steatosis in Histologic Sections by Supervised Learning

Hepatic steatosis was quantified from HE-stained images with the DL model generated in Aiforia Create. The model was first trained to detect liver parenchyma, including normal hepatocytes, macrovesicular hepatocytes, and microvesicular hepatocytes. The algorithm excluded background, tissue artefacts, blood vessels, and other features that do not belong to the liver parenchyma. The second algorithm was trained to recognize microvesicular and macrovesicular steatosis in the liver parenchyma, and eventually these two algorithms were combined to generate the final model (Supplemental Table S1).

The development of both algorithms followed a similar workflow. The model development started with labeling the training data, including 77 WSIs of HE-stained mouse liver samples from wild-type mice and mice deficient in hydroxysteroid (17β) dehydrogenase 13 and deficient in hydroxysteroid (17β) dehydrogenase 12, as well as the 24 WSIs of HE-stained mouse liver samples from C57Bl mice with a Western diet. The annotated training set (*n* = 101) was not used for validation. The representative areas of both morphologic features were selected visually, and the training areas and feature labels were drawn with the pen-tool in Aiforia by one person. The annotated training areas were selected to represent typical features of microvesicular steatosis and macrovesicular steatosis, annotation areas varied in size and color to offer a variety of examples for training, and annotations were drawn with versatile magnification views. The tissue was considered to contain macrovesicular steatosis if the single lipid droplets were bigger than the average size of nucleus and microvesicular steatosis if the lipid droplets were smaller than average size of the nucleus. During the training of the model, unclear regions were compared with Oil Red O–stained sections from the same liver sample to confirm the presence of fat. Annotation areas varied in size between 25 and 250 μm^2^, and all together 846 areas were annotated (Supplemental Table S1). If one training area included parenchyma, microvesicular steatosis, and macrovesicular steatosis, borders were drawn and different areas were labeled only to one class. The DL model was trained on the basis of these annotations, and the resulting model was evaluated visually. On the basis of the evaluation, parameters were optimized, annotations were edited, and new annotations were generated, followed by a new model version. Overfitting was minimized by training the model with a large and diverse data set, by using data augmentation, and by having separate testing data for validation. The training was performed through an iterative process, and the final DL model was a result of model version 11, trained with 2000 iterations (Supplemental Table S2).

### Validation of the DL Model Quantifying Hepatic Steatosis in Histologic Sections

To evaluate the performance of DL model, the annotations that formed the ground truth were compared with the analysis results of the artificial intelligence model both statistically in Aiforia and visually.

Eventually, the performance of the artificial intelligence model was validated using 20 HE-stained WSIs, which were not used for training, and the results obtained with the model were compared with the classification provided by two experienced pathologists (S.B. and N.S.). The pathologists provided an evaluation of the histologic features of steatosis by providing the percentage of hepatocytes having features of microsteatosis and macrosteatosis. In addition, a third experienced liver pathologist (E.-M.B.) performed pixel-level validation in Aiforia Create.

### Statistical Analysis

Statistical analyses were performed using GraphPad Prism software version 8.4.2 (GraphPad Software, La Jolla, CA). The Shapiro-Wilk test was used for testing the normality of the data. Differences between the two groups were evaluated using the two-tailed *t*-test and *U*-test based on if the data were normally distributed. Correlation was tested by using a Pearson correlation coefficient (*r*). *P* < 0.05 was considered significant.

## Results

### The DL Model Recognizes the Parenchyma and Is Able to Differentiate Microvesicular and Macrovesicular Steatosis

**Figure 1:**
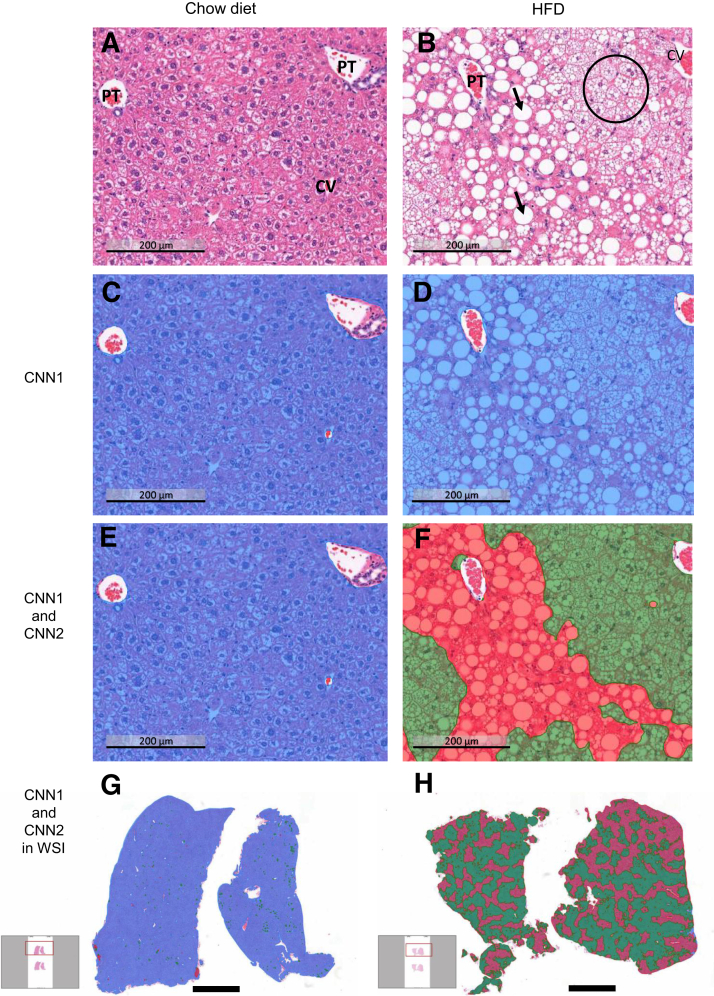
**A:** A representative image of mouse liver formalin-fixed, paraffin-embedded specimen stained with hematoxylin-eosin, including portal tract (PT) and central vein (CV). **B:** When liver steatosis develops, macrovesicular lipid droplets (**arrows**) and microvesicular steatosis (**circle**) are present. **C** and **D:** The model detects parenchymal tissue and excludes the white background, fixation artefacts, and vessel structures [convolutional neural network (CNN) 1]. **E** and **F:** Representative image showing that the model is able to detect and quantify the area of tissue containing microvesicular and macrovesicular steatosis (CNN2) inside parenchyma. **G** and **H:** The model enables analysis of a whole slide image. Scale bars: 200 μm (**A**–**F**); 2 mm (**G** and **H**). HFD, high-fat diet; WSI, whole slide image.

The model identified the parenchyma precisely, and efficiently excluded white background and artefacts in the tissue. It also excluded the blood vessels and other similar features that were not part of the liver parenchyma (Figure 1, C and D). As expected, HE staining of liver tissue showed lipid droplets in the steatotic livers of the HFD-fed mice (Figure 1, A and B). The model accurately detected and quantified the area of tissue containing lipid droplets, typically including both microvesicular and macrovesicular steatosis (Figure 1, E and F), enabling analysis of a whole slide image (Figure 1, G and H).

Image analysis enables a visual examination of the spatial location of steatosis. Images of the validation data, which were composed of mouse liver samples with HFD-induced steatosis, indicated that macrovesicular steatosis was usually located in the vicinity of the portal tract, whereas microvesicular steatosis was primarily present close to the central vein.

### The Model Quantifies Microvesicular and Macrovesicular Steatosis

The verification tool in the Aiforia Create was utilized to detect false-positive and false-negative areas of microvesicular and macrovesicular steatosis tissue and their sum (ie, total error per training area). The results indicated that the model learned the ground truth with high precision (>98%) and sensitivity (>99%) (Table 1).

**Table 1:** Performance Metrics of the Model

Metric	CNN1: parenchyma	CNN2: steatosis		
		Total	Microsteatosis	Macrosteatosis
Precision, %	99.64	98.42	98.23	98.81
Sensitivity, %	99.89	99.38	99.45	99.23
F1 score, %	99.76	98.90	98.84	99.02
Total area error, %	0.13	0.03	0.02	0.01
Error (false positive/false negative), %	0.47 (0.37/0.11)		2.34 (1.79/0.55)	1.97 (1.19/0.77)
False positive, %	0.10	0.01		
False negative, %	0.03	0.00		

Total area errors, precisions, and sensitivities per each CNN. Total area errors are the sum of false-positive and false-negative detection of the model compared with the ground truth (namely, the total errors per training areas in each CNN of the artificial intelligence model).

CNN, convolutional neural network.

In addition, a third experienced liver pathologist (E.-M.B.) performed a pixel-level validation in Aiforia Create. For this, a separate expert drew 61 representative validation areas in 39 WSIs, and the validator classified the tissue area into parenchyma, microvesicular steatosis, or macrovesicular steatosis. When compared with this evaluation by a pathologist, the sensitivity of the model was 97% and precision was 89% with total area error of 8.39%, showing a good performance by the model (Supplemental Table S3).

### The Performance of the Artificial Intelligence Model Replicates the Independent Evaluation by Pathologists

**Figure 2:**
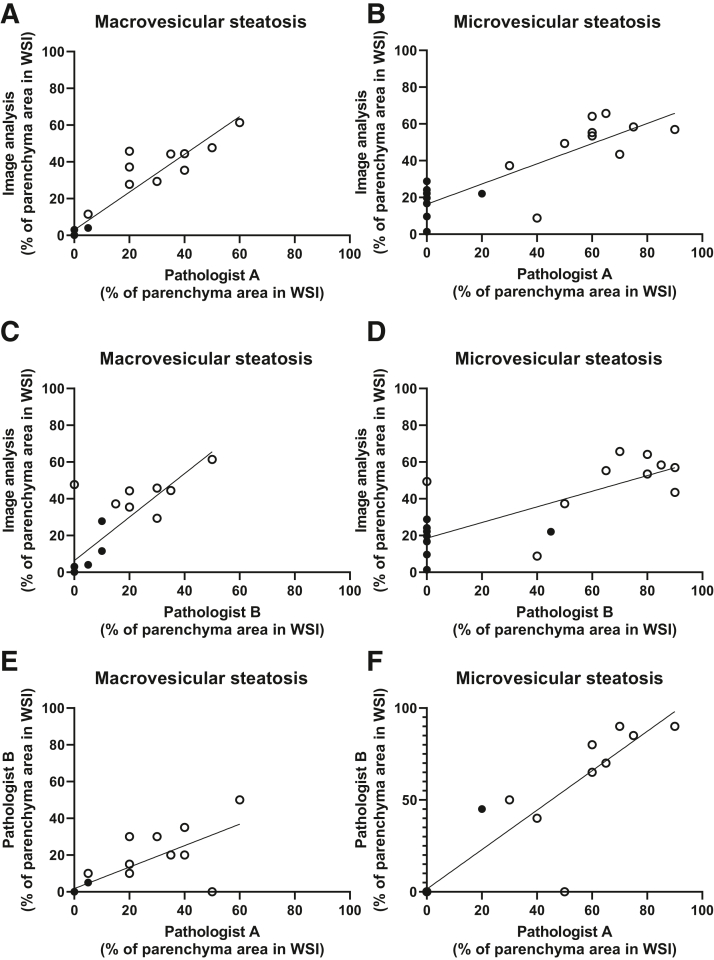
The correlation between the result obtained by the deep learning model and two pathologists (S.B. and N.S.). The validation set included hematoxylin-eosin–stained whole slide images (WSIs) from the liver of wild-type mice on normal chow (closed circles) and on a high-fat diet intervention (open circles). A strong positive correlation was observed between the image analysis and the pathologists' evaluation of both macrovesicular steatosis [*r* = 0.943 (*P* < 0.001; **A**), *r* = 0.823 (*P* < 0.001; **C**), and *r* = 0.771 (*P* < 0.001; **E**)] and microvesicular steatosis [*r* = 0.838 (*P* < 0.001; **B**), *r* = 0.762 (*P* < 0.001; **D**), and *r* = 0.914 (*P* < 0.001; **F**)]. *n* = 10 wild-type mice on normal chow and on a high-fat diet.

Part of the validation data (including 20 HE-stained WSIs of mouse liver samples from wild-type mice, half of which were kept on an HFD for 12 weeks) was further used to compare the performance of the artificial intelligence model with the staging provided by two experienced human pathologists (S.B. and N.S.). This was done by evaluating the proportion of cells containing microvesicular or macrovesicular steatosis by both the model and the pathologists. The Pearson correlation coefficients between pathologist A and the image analysis were 0.943 (*P* < 0.001) in macrovesicular steatosis (Figure 2A) and 0.838 (*P* < 0.001) in microvesicular steatosis (Figure 2B). The corresponding correlations between pathologist B and image analysis were 0.823 (*P* < 0.001) and 0.762 (*P* < 0.001), respectively (Figure 2, C and D). The correlation between the analyses by pathologist A and B was 0.771 (*P* < 0.001) in macrovesicular steatosis (Figure 2E) and 0.914 (*P* < 0.001) in microvesicular steatosis (Figure 2F).

### Image Analysis Results Performed by the DL Model Correlate with Other Measurements of Fat Content in the Mouse Liver

**Figure 3:**
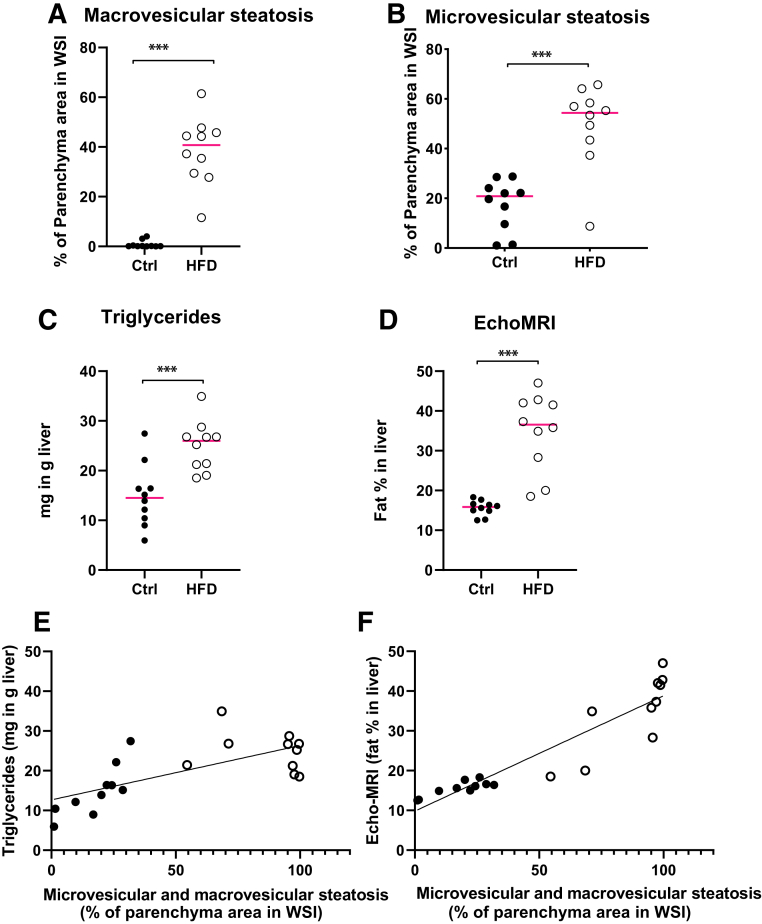
**A** and **B:** The image analysis performed with the model shows significant increase in the microsteatosis and macrosteatosis in the liver of mice on a high-fat diet (HFD) compared with a chow diet. **C** and **D:** The analysis by the deep learning (DL) model correlated well with the triglyceride concentration measurement in liver homogenate (**C**), and with the liver fat content analyzed by an EchoMRI *ex vivo* (**D**). **E** and **F:** The Pearson correlation coefficient between microvesicular and macrovesicular steatosis, defined by the DL model and triglyceride measurements, was 0.690 (*P* = 0.0008; **E**), and between microvesicular and macrovesicular steatosis and tissue EchoMRI, the correlation was 0.924 (*P* < 0.0001; **F**). **Red line** indicates mean value (**A**, **C**, and **D**) or median value (**B**) (data not normally distributed). ∗∗∗*P* < 0.001. Ctrl, control; WSI, whole slide image.

**Figure 4:**
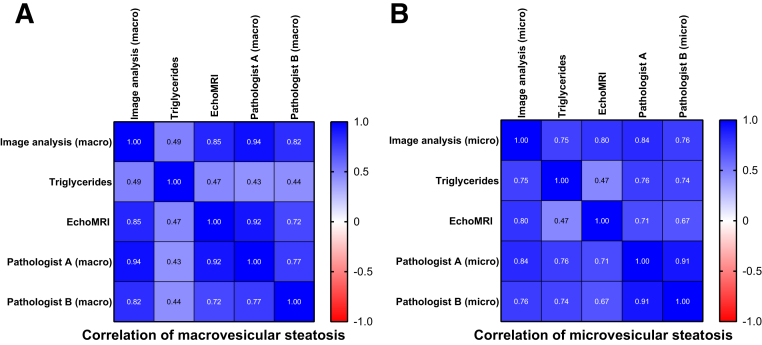
The correlation matrix between the various analyses of the fat content (Pearson correlation coefficient). The image analysis result and pathologists' (S.B. and N.S.) evaluation differentiate microvesicular (micro) and macrovesicular (macro) steatosis, but triglyceride and EchoMRI measurements assess the whole fat content. **A:** In the case of macrovesicular steatosis, both pathologists' evaluation and the EchoMRI measurements correlate well with the deep learning (DL) model, but the correlation with the total liver triglycerides is less strong. **B:** In relation to the presence of microvesicular steatosis, the pathologists' evaluation and both the EchoMRI and the triglyceride measurements show correlation with the DL model; however, the correlation between the EchoMRI and triglycerides is less strong.

The data obtained with the validated DL model showed a marked increase in both macrovesicular and microvesicular steatosis in mice on an HFD compared with the chow diet (Figure 3, A and B). Furthermore, a similar increase in fat content was observed in a whole liver analysis using EchoMRI *ex vivo* (Figure 3D) and by measuring the triglyceride concentration in tissue homogenate (Figure 3C) in corresponding liver samples. Furthermore, the calculated Pearson correlation coefficient between EchoMRI and the DL model was 0.92 (Figure 3F), and between triglyceride measurement and image analysis, the correlation coefficient was 0.69 (Figure 3E). A correlation matrix for all analyses performed is presented in Figure 4.

## Discussion

In this study, an automatic image analysis method based on deep learning was developed to quantify microvesicular and macrovesicular steatosis in WSIs of HE-stained sections of mouse liver, and the performance of the model was evaluated using specimens with various degrees of steatosis. The DL model detected the parenchyma with high confidence and quantified the tissue areas, including microvesicular and macrovesicular steatosis, as evidenced by the high correlation with the results obtained by experienced pathologists. Further reference data for the method validation were obtained from liver fat content measurements performed with an EchoMRI and from the measurement of the triglyceride concentration in liver homogenates. All the analyses indicated a reliable and robust performance by the model.

The advantage of the developed DL model is that, in addition to identification of macrovesicular steatosis, the model enables the quantitation of microvesicular steatosis. This is an important hepatic manifestation to be identified and quantified in various experimental mouse models, because of its potential role as a marker of hepatotoxicity.^6^^,^^22^ There are a few image analysis algorithms that are able to define microvesicular steatosis in mouse models. However, the accuracy of the detection of microvesicular steatosis in those algorithms has been questionable,^23^ or the method is based on segmenting individual lipid droplets in microvesicular steatosis.^16^

The DL model developed in this study quantifies the tissue area containing microvesicular and macrovesicular steatosis as opposed to quantifying individual lipid droplets. This approach is considered to be closer to the steatosis grading system used by the pathologists, where the proportion of steatosis is assessed by the evaluator as the percentage of the total area affected by steatosis.^12^^,^^24^ Recently, DL models for automated assessment of liver steatosis have been developed,^13^, ^14^, ^15^^,^^25^ which are trained with clinical patient data, whereas the DL model developed in this study is designed for preclinical studies. Although the histologic features of liver steatosis are somewhat similar in rodents and humans, the applicability of our model in human samples for research proposes has to be experimentally determined. Also, the robustness of the model for WSIs from other scanners is to be tested.

Mouse liver tissue is structured into lobes, which are divided in lobuli and further subdivided into zones. The zonal location of steatosis might reflect the origin of disease, and therefore, there is a great interest in developing methods to further define the spatial appearance of both microvesicular and macrovesicular steatosis in mouse liver.^26^ As far as we know, no such DL-based automatic analysis methods are currently available. However, the present model enables visualizing with pseudocolors the spatial appearance of the areas defined as including microsteatosis and macrosteatosis.

The model was developed with a software package including a licensing fee. The software allows users to train their own algorithms independently of the software provider. Also, the data, parameters, and ground truth used can be shared with other investigators for the reproduction of the DL model and replication of the results. When using supervised deep learning for image analysis, annotating the data requires histologic knowledge and the result is, to some extent, operator dependent. Therefore, interoperator and quantitative biochemical validation are of great importance. In addition, in histology-based techniques, only cross-sections can be analyzed, and comparison to methods applied to the whole biopsy is important for validation. One approach is to compare the performance of the histologic DL model with well-known biomarkers of the disease.^27^ In this study, the image analysis result reliably reflects the concentration of triglycerides measured from the tissue homogenates and the whole liver fat content analyzed by an EchoMRI *ex vivo*. The EchoMRI analysis used in this study is a novel method for such comparison, and the correlation between the EchoMRI and the image analysis by the DL model was good (Pearson *r* = 0.92). The concentration of triglycerides measured in liver homogenates differed slightly from other measurements, which is likely due to the technical challenges in extracting lipids from the liver tissue.

The results of the proposed DL model were in good concordance with the pathologists' evaluation. However, the discrimination of microvesicular steatosis from general ballooning and degeneration is still challenging, and it is notable that quantification of low level of microvesicular steatosis differed between the pathology evaluation and that performed by the DL model. The pathologists did not consider significant microsteatosis to be present in several cases where the DL model identified 0% to 30% of the areas to include signs of microvesicular steatosis. Interestingly, the triglyceride and EchoMRI measurement showed a good correlation with the DL model also in samples with a low level of lipid accumulation, indicating that the biomarkers used and the DL model developed are sensitive markers for detecting low levels of fat accumulation. The high sensitivity of the method developed can be considered a beneficial feature in analyzing, for example, effects of pharmaceutical and genetic interventions in preclinical animal models.

In addition to the diet-induced NAFLD models, various genetically modified mouse models present features of NAFLD.^19^^,^^20^^,^^28^ Thus, it was considered important to train a model with both genetically modified mice presenting with steatosis on a chow diet, as well as using HFD intervention models. This approach is expected to increase the suitability of this DL model for various data sets. Furthermore, the method allows for studying large sample cohorts with reduced hands-on time and increase the reproducibility of the data. In conclusion, the present study developed a DL image analysis model to quantify microvesicular and macrovesicular steatosis in mouse liver, applicable for studying NAFLD phenotype in models with dietary interventions as well as in genetically modified mouse models and a combination of the two.
