# Supplementary material for: Deep Learning–Based Image Analysis of Liver Steatosis in Mouse Models
Source: Am J Pathol. 2023 May 24;193(8):1072–80. doi: 10.1016/j.ajpath.2023.04.014 (PMC12178343; doi:10.1016/j.ajpath.2023.04.014)
Supplement: Supplemental Table S1 [file mmc1.docx]

**Supplementary Table S1. Ground truth (Definition of layers)**

| **C**NN | **Definition** | **Excluded features** | **Total area of the training regions** | **Total Area of the annotated region:** | **Total number of images used** |
| --- | --- | --- | --- | --- | --- |
| Liver parenchyma | Parenchymal tissue, including normal, macrovesicular and microvesicular hepatocytes | White background, blood vessels, tissue artefacts, tissue holes | 202.338mm^2^ | 54.914mm^2^ | 105 |
| Macrovesicular steatosis | Liver tissue containing macrovesicular lipid droplets (droplets>6µm) | Liver tissue that not include lipid droplets | 478.491mm^2^ | 2.355mm^2^ | 97 |
| Microvesicular steatosis | Liver tissue containing microvesicular lipid droplets (droplets <6µm) | Liver tissue that not include lipid droplets | 478.491mm^2^ | 4.754mm^2^ | 97 |
