# Supplementary material for: Deep Learning–Based Image Analysis of Liver Steatosis in Mouse Models
Source: Am J Pathol. 2023 May 24;193(8):1072–80. doi: 10.1016/j.ajpath.2023.04.014 (PMC12178343; doi:10.1016/j.ajpath.2023.04.014)
Supplement: Supplemental Table S2 [file mmc2.docx]

**Supplementary Table S2. CNN details (Aiforia model hyperparameters)**

CNN1 is a parent layer and CNN2 is a child layer. They are independent layers connected to each other by filtering.

|  | | **CNN1: Parenchyma** | **CNN2: Steatosis** |
| --- | --- | --- | --- |
| **Classes** | | Parenchyma | Micro, macro |
| **Type (semantic segmentation)** | | Region | Region |
| **Complexity** | | Very Complex | Very Complex |
| **Field of View** | | 100µm | 90 µm |
| **Training parameters** | **Weight decay** | 0.0001 | 0.0001 |
|  | **Mini-batch size** | 40 | 40 |
|  | **Mini-batches per iteration** | 20 | 20 |
|  | **Iterations Without progress** | 500 | 500 |
|  | **Initial learning rate** | 0.1 | 0.1 |
| **Image augmentation** | **Scale (min/max)** | -10/10 | -10/10 |
|  | **Aspect ratio** | 10 | 10 |
|  | **Maximum shear** | 10 | 10 |
|  | **Luminance (min/max)** | -20/20 | -10/10 |
|  | **Contrast (min/max)** | -20/20 | -10/10 |
|  | **Max with balance change** | 5 | 5 |
|  | **Noise** | 1 | 1 |
