# Supplementary material for: Deep Learning–Based Image Analysis of Liver Steatosis in Mouse Models
Source: Am J Pathol. 2023 May 24;193(8):1072–80. doi: 10.1016/j.ajpath.2023.04.014 (PMC12178343; doi:10.1016/j.ajpath.2023.04.014)
Supplement: Supplemental Table S3 [file mmc3.docx]

**Supplementary Table S3.** **Pixel-level validation by external validator**

The pixel-level validation by external validator was performed only to the second layer.

|  | **Total** | **Micro** | **Macro** |
| --- | --- | --- | --- |
| **Precision** | 89.29% | 90.90% | 87.93% |
| **Sensitivity** | 96.60% | 98.25% | 95.20% |
| **F1 Score** | 92.80% | 94.43% | 91.43% |
| **Total area error** | 8.39% | 2.96% | 5.43% |
| **Error (FP/FN) %** |  | 11.58 (9.84/1.75) | 17.86 (13.06/4.80) |
| **False positive** | 3.24% | | |
| **False negative** | 0.95% | | |
